# DNA Vaccination: Using the Patient's Immune System to Overcome Cancer

**DOI:** 10.1155/2010/169484

**Published:** 2010-12-16

**Authors:** Georg Eschenburg, Alexander Stermann, Robert Preissner, Hellmuth-Alexander Meyer, Holger N. Lode

**Affiliations:** ^1^Experimental Oncology Group, Department of Pediatrics, Charité-University Medicine Berlin, 13353 Berlin, Germany; ^2^Structural Bioinformatics Group, Institute for Physiology, Charité-University Medicine Berlin, 14195 Berlin, Germany; ^3^Department of Urology, Charité-University Medicine Berlin, 10117 Berlin, Germany; ^4^Department of Pediatric Hematology and Oncology, University of Greifswald, 17475 Greifswald, Germany

## Abstract

Cancer is one of the most challenging diseases of today. Optimization of standard treatment protocols consisting of the main columns of chemo- and radiotherapy followed or preceded by surgical intervention is often limited by toxic side effects and induction of concomitant malignancies and/or development of resistant mechanisms. This requires the development of therapeutic strategies which are as effective as standard therapies but permit the patients a life without severe negative side effects. Along this line, the development of immunotherapy in general and the innovative concept of DNA vaccination in particular may provide a venue to achieve this goal. Using the patient's own immune system by activation of humoral and cellular immune responses to target the cancer cells has shown first promising results in clinical trials and may allow reduced toxicity standard therapy regimen in the future. The main challenge of this concept is to transfer the plethora of convincing preclinical and early clinical results to an effective treatment of patients.

## 1. Introduction

### 1.1. Immunotherapy and Cancer

Cancer is a leading cause of death and is responsible for a magnitude of all disease-related deaths worldwide [1, 2]. Standard cancer therapy includes intensive chemo- and/or radiotherapy able to effectively eradicate cancer cells but with the disadvantage of severe side effects. Additionally, many cancers are diagnosed at an advanced tumor stage, where standard therapy has its limitations and is only able to cure low numbers of patients. Vaccination against cancer is a promising approach to induce the immune system to specifically target the tumor cells. 

However, the successful use of cancer vaccines is dependent on several problems that need to be overcome. Advanced tumor progression often leads to immune suppression; patients are weakened by previous therapies and aging [3]. In mouse tumor models, there are indications that young mice are better protected against a lethal tumor challenge showing improved primary immune responses than older mice making the use of vaccines in patients at an advanced age challenging because the thymus stops producing naïve T cells with age [4, 5]. 

The fact that the status of the patient's immune system is critical for the ability to develop an effective antitumor immune response is supported by the correlation between the amount of tumor-infiltrating lymphocytes with a favourable prognosis [6, 7]. In contrast, patients who are chronically immune suppressed as a result of therapy or other reasons have an unfavourable prognosis [8, 9]. 

The challenge is to amplify the patients' own immune response and translate it into a long-lasting memory without induction of unmanageable autoimmunity in order to protect against metastasis in the future. 

One approach is the use of whole inactivated tumor cells as a source of antigen based on promising results in mouse models [10]. The biological background to this strategy is the presence of tumor-specific or tumor-associated antigens expressed by the cells used for vaccination and the malignant target cells. Many antigens involved in effective cell-based vaccination strategies were identified, characterized, and found to be also expressed on normal cells. The expression is often compartmentalized in distinct tissues and is frequently of a significantly lower magnitude than on the cancer cells; however, this fact always bears a risk of autoimmunity. Therefore, the ideal cancer vaccine targets tumor-specific antigens expressed exclusively on tumor cells or tumor-associated antigens without harming normal cells expressing the same antigen, a problem which is hard to solve. An important key to success of cancer vaccines is to break self-tolerance, since tumor-associated antigens are self-antigens overexpressed by tumor cells. This challenge involves the use of distinct prime-boost strategies with different formulations of the tumor-associated antigen used for vaccination. This includes peptide- or protein-based antigens or delivery with viral vectors, which need to be used in combination in order to elicit measurable immune responses [11, 12]. Promising results were obtained with the use of self-replicating RNA and DNA vaccines which were able to break tolerance against tumor-associated self-antigens involving pathways of innate antiviral immunity. These vaccines enhance the immunogenicity and production of antigen-specific antibodies and CD8^+^ T cells without negative side effects. Although the production of antigen was not increased in comparison with conventional DNA vaccines, it is likely that the efficacy of the self-replicating vaccines was associated with caspase-dependent apoptotic cell death of transfected cells and a subsequent uptake of these cells by dendritic cells (DCs) [13–15].

Application of xenogeneic tumor-associated antigens is another interesting strategy to overcome some of the obstacles mentioned above. The magnitude of an antitumor response is clearly improved by using a tumor-associated antigen obtained from a different species sharing critical epitopes flanked by xenogeneic protein sequences further stimulating the antitumor response [16–19]. The delivery of such xenogeneic tumor-associated antigens by DNA vaccination may be a promising venue to the design of a successful strategy.

### 1.2. DNA Vaccination

Historical observations that transfer of foreign DNA by different *in vitro* and *in vivo* techniques led to expression of antigen were the basis for the generation of DNA vaccines [20]. DNA vaccines can consist of tumor-specific or tumor-associated antigens (TAAs) and additional immune-stimulatory factors cloned into a bacterial plasmid downstream of an appropriate eukaryotic promoter for strong and stable expression. 

TAAs used for cancer vaccines are exogenous viral antigens expressed by virus-induced cancers, tumor-restricted antigens also called neoantigens, tumor-associated differentiation antigens that are only expressed in specific tissues, or generally expressed antigens that are overexpressed in cancer cells. 

Cancer vaccines include MHC class I and class II epitopes, multiple TAAs to effectively target the whole inhomogeneous tumor population to decrease the risk of immune escape, and can contain TAAs that correspond to proteins involved in tumor transformation [21]. DNA minigenes are a special type of DNA vaccines harbouring only short antigen epitopes which can efficiently induce a cytotoxic T cell (CTL), B-cell or T-helper cell response. They are as effective as whole cDNA vaccines but without the risk of introducing a functional cDNA with possible devastating consequences [22–25]. This is especially important if antigens that are used can function as oncogenes. If whole cDNA vaccines are favoured, rearranged or mutated sequences were shown to be useful for full immunological activity without the risk of negative properties of the functional protein [26, 27]. DNA vaccines have many advantages if compared with classical vaccines; they combine the diversity of possible TAAs expressed on whole tumor cells or subunit vaccines with the efficiency of *in vivo* antigen synthesis and presentation able to induce both cellular (CD4^+^ and CD8^+^ cells) and humoral immune responses [28]. 

The risks of DNA vaccines are limited [29]. Several groups demonstrated that cancer vaccines can be effective in induction of specific immunity against cancer-associated antigens without negative side effects like integration of plasmid DNA into the host genomes or induction of pathogenic anti-DNA antibodies [30–38]. The results in animal models and initial clinical trials are promising but so far have not resulted in a successful, standardized translation into the clinic, emphasizing the enormous differences between animal models and patients. Possible reasons may be related to the compromised immune system of the cancer patients after their chemo- and/or radiotherapy. Additionally, tumors develop mechanisms to escape the immune system, such as the loss of MHC class I molecules or antigen, so they cannot be recognized by CTLs [39–42]. Other mechanisms are the occurrence of regulatory T cells that negatively influence the induction of anti-tumour responses, systemic defects in immune cells, secretion of immunosuppressive cytokines, resistance to apoptosis, and many more [43, 44], which need to be addressed.

### 1.3. Effective Activation of the Patient's Immune System by DNA Vaccines

DNA vaccines capable to activate the patient's immune system to effectively target cancer need the activation of effector cells that are able to kill the tumor or can indirectly trigger a cascade that subsequently lead to its eradication. Naïve T cells are a basic part of this complex system which are activated if they get two independent signals. The first signal is provided by binding of a specific antigen-MHC class I complex to its T cell receptor (TCR). The second costimulatory signal is diverse in nature and may consist of CD40 expressed on antigen-presenting cells (APCs) or soluble factors such as cytokines (IL-2). To increase the immunogenicity of tumor cells, they were manipulated to express costimulatory molecules and/or cytokines thereby significantly enhancing the induction of an immune response [45, 46]. 

The delivery system used for the application of anticancer vaccines also plays an important role in increasing the amplitude of an immune response. Plasmid-based DNA vaccines can be applied with ballistic delivery (gene gun) [47], liposomal or microsphere encapsulation [48] incorporated in bacterial or host cell carriers [49, 50], or by electroporation [51–54]. The latter technique is used efficiently in preclinical and clinical trials of melanoma and prostate cancer. Using bacteria as DNA vaccine vehicles bears the advantage of efficient stimulation of the innate immune system through the recognition of lipopolysaccharides (LPSs) in the bacteria outer membrane. LPSs stimulate APCs by binding to Toll-like receptor 4 (TLR4) which subsequently supports an efficient activation of T cells, directly activates natural killer cells (NK cells), or leads to an increased lifetime of antigen-specific T cells [12, 55]. Unmethylated CpG motifs included in DNA of bacterial origin have an additional immune stimulatory effect on cells of the innate immune system. Binding of CpGs to Toll-like receptor 9 (TLR9) expressed on DCs, NK cells, or monocytes/macrophages leads to further maturation and activation of these cells and subsequent secretion of proinflammatory cytokines of the Th 1 type including IL-12, TNF-*α*, IFN-*α*, and IFN-*γ* and the upregulation of costimulatory molecules such as CD80 and CD86 on APCs [56–58]. The dependency of TLR9 activation by CpGs is challenged by recent observations indicating that the DNA sugar backbone is also crucial for either activation or inhibition of TLRs by DNA. Natural DNA activates TLRs with phosphodiester (PD) backbone independent of CpGs in contrast to synthetic phosphorothioate- (PS-) modified DNA, which is TLR antagonistic. In the latter case, CpG motifs can transform the antagonistic PS-modified DNA to a strong activator of TLRs restricting the dependency of CpGs on TLR activation to this special case [59, 60]. Furthermore, the detection of foreign non-CpG DNA is also mediated by TLR-independent sensors leading to the expression of interferon genes and induction of innate immunity. One candidate sensor called DNA-dependent activator of interferon regulatory factors (DAI) was shown to directly interact with DNA in interplay with the interferon regulatory factor 3 transcription factor (IRF3) leading to a release of interferon-*β* in a TANK-binding kinase 1- (TBK1-) dependent manner [61–63]. Another crucial factor in the activation of the TBK1 pathway seems to be STING (stimulator of interferon genes) [64]. This novel independent pathway shows that AT-rich DNA can also serve as a template for RNA polymerase III leading to a transcription into double-stranded RNA (dsRNA). dsRNA subsequently acts as a ligand for the potential cytosolic DNA sensor RIG-I (Retinoic acid-induced gene I) and to a production of type I interferons [65, 66].

### 1.4. Presentation of Antigens Encoded in DNA Vaccines

Based on the DNA vaccine delivery system and the DNA design of the antigen sequences, there are at least three different mechanisms as DNA vaccines can be processed and presented *in vivo*. First, DNA vaccines can directly lead to production of the antigen by somatic cells like keratinocytes or myocytes. These cells share a poor capability to directly present processed antigen to immune cells by MHC class I and II molecules. Therefore, this mechanism is considered to play only a subordinate role. Second, the production of antigen by somatic cells may result in effective presentation to the immune system by a mechanism called “cross-priming”. Antigen may be released from the site of production travelling to the draining lymph node, where it is taken up by APCs processed and presented to T cells [67, 68]. Third, DNA vaccines also lead to the production and direct presentation of antigen by professional APCs. For this purpose, the normal infection pathway of intracellular bacteria, for example, attenuated *Salmonella thyphimurium*, can be used for oral DNA vaccination. The bacteria enter the host through the gastrointestinal tract after oral gavage and move through the M cells that cover the Peyer's patches (lymph nodes) of the gut. From there, they enter APCs like macrophages or DCs by phagocytosis. In the APCs, the bacteria die, delivering multiple copies of the vaccine DNA that can encode for antigen to the phagosome or cytosol [69, 70].

### 1.5. Processing DNA-Encoded Antigen

The activation of CTLs which are key players in DNA vaccine-mediated tumor immunity is induced by degradation of protein components into smaller peptides and presentation of antigen by APCs [71, 72]. 

Processed antigens can be either presented by the endogenous or the exogenous pathway. CD8^+^ T cells, the precursors of CTLs, are in general activated by intracellular pathogen-derived antigens of 8–10 amino acids length which are presented by MHC class I molecules (endogenous pathway) [73, 74]. In contrast, CD4^+^ T-helper cells generally recognize exogenous antigens presented as 12–15 amino acid long peptides bound to MHC class II molecules. Upon activation, T-helper cells secrete cytokines, which is crucial for the induction and maintenance of immunologic memory [75–78]. If antigens are transported to the cytoplasm or proteins are produced endogenously, they are degraded by the proteasome into small peptide fragments. These peptides are then transported by TAP1 and TAP2 into the endoplasmatic reticulum (ER) and bind to a dimer consisting of an MHC class I molecule and *β*_2_-microglobulin. MHC class I antigen complexes are then transported to the cell surface, where they are presented. The trimolecular complex can be recognized by CD8^+^ cytotoxic T cells. This mechanism is important for induction and activation of antigen-specific CD8^+^ T cells by APCs and for the effector function of CD8^+^ cytotoxic T cells after trimolecular complex recognition on the tumor cell resulting in subsequent target cell lysis [79–82]. Presentation of antigens via the endogenous pathway dominantly leads to activation of Th 1 cells and CTL responses, whereas the exogenous pathway leads to the activation of Th 2 cells and the production of antibodies [83, 84]. DNA vaccines can increase the Th 1 immune response and the levels of immunoglobulins by directly inducing the expression of interferons, IL-12, IL-18, or TNF-*α* [85]. The activation of the exogenous pathway is usually insufficient to prevent tumor growth in animal models of cancer but is in contrast preferable in the case of protection against extracellular pathogens or in targeting chronic infections. 

The introduction of specific sequences to the antigen like an N-terminal ubiquitination signal can further enhance the induced CTL response, preferentially of the Th 1 type [86]. It is possible to increase the protection against virus or tumor challenge by directing the antigen to specific cell compartments like the proteasome leading to fast degradation and presentation of antigen to MHC class I molecules [87]. Ubiquitination of DNA minigenes probably leads to polyubiquitination of the cleaved peptide epitopes resulting in a more effective delivery of the minigene to the proteasome and an increase of the frequency of CTL precursors [24].

## 2. Melanoma

Malignant melanoma is a neuroectodermal solid tumor affecting predominantly Caucasians. More than 160.000 new cases were diagnosed in 2002 with an increasing incidence. Despite favourable survival rates in the developed countries of greater than 90% in early stages, around 40.000 deaths were caused by melanoma in 2002. In the US alone, more than 10.000 people will probably die of skin cancer in 2010, and the prognosis of stage IV melanoma remains poor (<20% 5 y EFS) [1, 88]. Melanoma is a highly immunogenic cancer and maybe the most prominent model for DNA vaccination and the development of tumor vaccines in general [89]. In patients, spontaneous complete melanoma remission is occasionally detected, a phenomenon mediated by endogenous CTLs and subsequent tumor rejection [90, 91]. A variety of melanocyte differentiation antigens were identified as tumor antigens and found to be highly expressed in melanoma cells. Consequently, these antigens were successfully used as targets for DNA vaccination, and their application in humans, dogs, and preclinical models are discussed in the following paragraphs [92, 93]. 

Based on the successful use of gp100, MART-1/Melan-A, and tyrosinase, several clinical trials were conducted or are still ongoing. In an initial study, patients with metastatic melanoma were immunized with human gp100 (hgp100) expressing naked plasmids showing no clinical or immunological responses, indicating that the delivery system and adequate costimulation play an important role for the success of this approach. For example, the use of fowl poxvirus encoding for hgp100 or hgp100 peptides was capable to break the self-tolerance against the gp100 tumor antigen [40]. Also the use of particle-mediated epidermal delivery (PMED) of hgp100 cDNA in combination with costimulatory granulocyte macrophage colony-stimulating factor (GM-CSF) into healthy skin of melanoma patients revealed the recruitment of DCs to the vaccination sites. Subsequently, a low but detectable antimelanoma immune response was observed [94]. A role for GM-CSF DNA as an adjuvant was established in a phase I/II trial using a DNA vaccine encoding for hgp100 and tyrosinase epitopes resulting in specific CD8^+^ responses in 42% of the treated melanoma patients [95]. Another strategy effectively break tolerance against self-antigens is the use of xenogeneic DNA vaccines. This is indicated by two recent phase I trials using mouse gp100 (mgp100) DNA vaccines alone or in combination with the human homologue. Melanoma patients immunized with the xenogeneic vaccines developed hgp100-specific and IFN-*γ*-secreting CD8^+^ T cells, and 30% of them showed an immune response [16, 96]. 

MART-1 is another melanoma antigen used in clinical DNA vaccination trials. In an early phase I study, 12 patients with resected melanoma received MART-1 plasmids, again without further adjuvant strategy. Immunological responses were not detectable [97]. Similar poor results were observed in nineteen patients with stage IV melanoma which were treated with a plasmid encoding T cell epitopes from MART-1 and tyrosinase by intranodal injection [98]. The vaccination approach induced an immune response in some of the patients but was not able to stop the progression of the disease. This again suggested that DNA vaccines have to be used in combination with adjuvants, cytokines, or in the context of distinct prime/boost approaches to increase the immune response for effective treatment of melanoma. 

Changing the application system from naked plasmid to a viral delivery system may not be sufficient to translate immune to clinical response demonstrated by clinical DNA vaccination trials using tyrosinase as antigen. Tyrosinase was used as single cDNA vaccine applied in stage II melanoma patients by recombinant modified vaccinia virus Ankara (MVA). There was a strong immune response against the virus, indicated by virus-specific CD4^+^ and CD8^+^ T cells and antibody titres, but no tyrosinase-specific T cells or antibodies were detected [99]. In two subsequent clinical trials, tyrosinase was delivered by vaccinia or fowlpox viruses which were applied to patients with advanced metastatic melanoma who also received systemic IL-2 [100]. Antityrosinase immunity was enhanced in some patients but without clinical benefit compared to effects expected for IL-2 alone.

Numerous DNA vaccination studies in animal models of melanoma demonstrated efficacy. Particular success was reported for xenogeneic strategies, viral delivery systems, and the use of IL-2 as an adjuvant [101]. In mouse models, efficacy of DNA vaccines encoding for all known melanoma-associated antigens was reported including gp100 (melanocyte protein 17/Pmel-17), GRP (gastrin-releasing peptide) [102], MAGE-1 (melanoma-associated antigen) [103], MART-1, MUC-18/MCAM [104], TRP-1 (tyrosinase-related protein-1/gp75), TRP-2, or tyrosinase. Also inhibitor of apoptosis proteins (IAPs) like ML-IAP (melanoma inhibitor of apoptosis protein) [105] and survivin [106] were used. In some approaches, less relevant antigens were used such as melanoma cell lines stably transfected to express viral antigens of hepatitis B virus or HPV (human papillomavirus) or human oncogenes like Mucin 1 (MUC-1) [107–109]. For vaccination studies in mice, usually the melanoma cell line B16 is used syngeneic to C57BL/6 mice leading to melanoma growth and metastasis serving as a model for the human disease [110, 111]. In view of the limited efficacy of DNA vaccines in clinical trials so far, these successful studies in murine models reflect the difficulty of the transfer of results from mice to man.

However, there are important lessons to be learned from animal models such as the important role of adjuvanticity and prime/boost schedules. The majority of vaccination studies in mice were done with gp100 as melanoma target antigen, and some results will be discussed here in more detail. In early studies, vaccination of mice by s.c. injection of plasmid encoding for hgp100 antigen alone or in combination with GM-CSF DNA was conducted in a B16 model transfected with hgp100 DNA (B16/hgp100) [112, 113]. Protection against melanoma challenge and reduction of established primary tumor growth was observed, and hgp100-specific CTLs and antibodies were found. However, immunity against mpg100 was poor. Changing the delivery system by incorporation of the plasmid DNA expressing hgp100 into liposomes was able to induce an mgp100 mediated protective immunity [114]. Vaccination induced a delayed primary tumor growth, a phenomenon which was also seen in another study using an mgp100 plasmid [115]. Vaccination of mice with attenuated *Salmonella typhimurium *(ST) transformed with mgp100 cDNA significantly reduced melanoma growth, an effect which was increased by IL-2 administration [116]. In the B16/hgp100 model, vaccination with hgp100 transformed ST was able to completely protect more than 70% of the vaccinated mice mediated by a strong anti-hgp100 CTL response [117]. The superiority of xenogeneic vaccination approaches became obvious in a study were hgp100 plasmids were applied via helium-driven DNA-gold complexes leading to a tumor protection in many of the mice which was mediated by specific CD8^+^ T cells with the typical side effect of autoimmune depigmentation [118]. A comparison of hgp100 and mgp100 full-length DNA and minigenes revealed that only the human constructs were able to induce a CD8^+^ dependent tumor protective immunity further strengthening the xenogeneic vaccination concept [119]. Tumor formation was completely prevented in more than 30% of the treated C57BL/6 mice when a hgp100 plasmid was used together with synthetic peptides of putative CTL epitopes, an effect which was not observed if plasmid or peptides were used alone [120]. The used study design also demonstrated a therapeutic effect of the combinatorial setting and a dependency on CD4^+^ and CD8^+^ T cells for melanoma protection. Codelivery of IL-12 DNA by direct injection into tumors was able to further increase the antitumorale effect induced by hgp100 DNA vaccination [121]. An autologous gp100 plasmid was able to break self-tolerance when a different mice model was used, showing that differences in genetic backgrounds are critical parameters for success of DNA vaccination [122]. DBA/2 mice were challenged with either mgp100 positive or negative syngeneic M3 melanoma cells leading to an mgp100-specific T-cell-mediated immune response and a protection against melanoma growth only if the mgp100 expressing cells were used.

Similar results were obtained using TRP-1/gp75 DNA vaccines in the B16 model in several syngeneic and xenogeneic settings. Murine TRP-1 (mTRP-1) expressing plasmids were not able to break self-tolerance against mTRP-1 in contrast to the human homologue which induced immunity against mTRP-1 and subsequent tumor protection and eradication [123]. Rejection of a lethal challenge with B16 melanoma cells was achieved with mTRP-1 encoded by a recombinant vaccinia virus again emphasizing that the adjuvanticity of the delivery system is important to break self-tolerance [124]. Boosting of DNA vaccination by application of monoclonal antibodies is another strategy used with a hTRP-1 DNA vaccine [125]. Lung metastases induced by B16 were significantly decreased if hTRP-1 DNA was used with TA99, an antibody targeting TRP-1, a synergistic effect which was not seen with vaccine or antibody alone. The TA99 antibody seemed to be responsible for the recruitment of TRP-1-specific CD8^+^ T cells to the tumor and subsequent tumor infiltration.

Also DNA vaccination using TRP-2 as a target revealed that vaccination of mice with murine TRP-2 (mTRP-2) using vaccinia virus as a delivery system was more effective than naked DNA injection [126, 127]. Again the xenogeneic concept was more effective since hTRP-2 DNA prevented the growth of B16 cells in the skin of treated mice by activation of CD4^+^ and CD8^+^ T cells [128]. Additional treatment with hTRP-2 after surgical resection of affected extremities also reduced the reoccurrence of local disease and the number of lung metastases. In summary, adjuvanticity, delivery systems, and prime/boost schedules are important factors to consider from preclinical models for the design of an effective DNA vaccination strategy in humans. The design of the antigen including signals for proteasomal degradation also plays an important role in vaccine efficacy. In this regard, fusion of mTRP-2 to ubiquitin facilitated proteasome-dependent degradation of antigen and subsequent presentation of epitopes to MHC-class I leading to the generation of mTRP-2-specific CD8^+^ T cells. These T cells were not only capable to protect against melanoma but also had a therapeutic effect on established melanomas [129]. Other strategies may enhance vaccine efficacy including lymphodepletion with cyclophosphamide and antigen fusion with heat shock proteins. The role of lymphodepletion in adoptive T cell transfer strategies has been demonstrated [130, 131]. Similar concepts may apply for DNA vaccines. Adenoviral delivery of hTRP-2 in combination with high-dose cyclophosphamide had a synergistic effect and improved the outcome of tumor-bearing mice [132]. Also fusion antigens are an interesting approach to further enhance immunogenicity. Fusion of heat shock protein 70 (Hsp70) to tumor antigens led to efficient delivery of antigen to APCs thereby breaking the immune tolerance against melanoma cells [133]. An Hsp70-mTRP-2 DNA vaccine was orally applied by transfected attenuated *S. typhimurium* strain SL3261 protecting more than half of the treated mice from a lethal challenge with B16 melanoma cells in a prophylactic setting and prevented or significantly reduced tumor growth in a therapeutic setting.

There are promising results with a xenogeneic DNA vaccination strategy in dogs with melanoma, which raise hope that this strategy will succeed in humans as well. Therapy with xenogeneic tyrosinase DNA vaccines was used in phase I trials of spontaneous advanced malignant melanoma in dogs, a disease very similar to human melanoma. Intramuscular injections with human tyrosinase (hTyr) plasmids significantly increased the expected survival of the dogs compared to matched historical controls, and one dog with stage IV disease had a complete clinical response [134]. In three of the nine treated dogs, tyrosinase-specific antibodies were induced by vaccination with hTyr DNA which partially reacted with the syngeneic canine tyrosinase, a phenomenon which correlated with the observed clinical response and possibly responsible for the tumor static effects and long-term survival of the dogs [135].

In summary, the human clinical trials led to promising results like activation of melanoma antigen-specific CTLs but have so far not resulted in significant improvements in outcome. Studies in small and large animals were nevertheless able to demonstrate efficacy of DNA vaccination against melanoma. Some critical parameters were identified including the delivery systems, adjuvants, and antigen design. In general, the use of xenogeneic antigens often showed better results in the treatment of melanoma by DNA vaccination, and the use of viral application methods and cytokines further increased immunogenicity. The consideration of the lessons learned from animal models for the design of DNA vaccination strategies may lead to an effective approach in the future.

## 3. Neuroblastoma

Neuroblastoma (NB) is the most common extracranial solid tumor in childhood. The prognosis is still poor, and the development of effective treatment strategies is one of the main objectives in pediatric oncology. Despite the development of innovative treatment strategies like passive immunotherapy with the anti-GD2 antibody ch14.18, the long-time survival rate especially of stage 4 tumor patients remains poor, ranging between 35 and 40% [136, 137]. The power of immunotherapy was demonstrated in a recent phase III trial combining ch14.18, GM-CSF, and IL-2 with standard therapy raising the hope to significantly increase the long time survival rate of high-risk patients in the near future. Patients who received the immunotherapy showed an improved outcome compared to standard therapy with a two-year event-free survival (EFS) of 66% and 46%, respectively [138]. 

In animal models, there are several promising results showing that DNA vaccination is able to protect mice from a lethal challenge with neuroblastoma tumor cells. Many results in this respect were generated using the syngeneic NXS2 neuroblastoma mouse model. This hybrid cell line expresses ganglioside GD2 which is highly expressed in NB and, as an established tumor marker, is used as a target in clinical trials of NB immunotherapy. 

The NXS2 model mimics the human disease in several aspects. It features spontaneous metastasis to bone marrow and liver after injection of the cells in syngeneic A/J mice, making this system an ideal model to study DNA vaccination [139]. One example is cyclic mimicking decapeptides of GD2 which were successfully used for DNA vaccination generating protection against tumor growth and a reduction of spontaneous liver metastases [140]. Codelivery of the cytokines IL-15 and IL-21 enhanced the induction of GD2 directed responses which increased the CD8^+^ T cell function. The effects were NK cell as well as CD4^+^ and CD8^+^ T cell mediated indicating the involvement of innate and adaptive immune responses [141]. A plasmid that encoded for the secreted form of HuD was able to induce a strong and specific anti-HuD response in a similar mouse model using A/J mice with the Neuro2a NB cell line. Mice that were challenged with constitutively HuD-expressing Neuro2a cells were protected against tumor growth after immunization with HuD DNA vaccine but showed no signs of neurological disease induction [142]. 

The neuroblastoma antigen tyrosine hydroxylase (TH) is another promising candidate for immunotherapy of neuroblastoma. Tyrosine hydroxylase is involved in the first step of catecholamine biosynthesis, a unique feature similar to melanin biosynthesis in melanoma, involving enzymes restricted to the tumor tissue therefore providing tumor-associated antigens.

Prophylactic and therapeutic vaccination with murine TH cDNA or TH minigenes was able to protect against tumor growth after delivering plasmid DNA by oral gavage of attenuated *S. thyphimurium* to the mice. The used expression vector contained a mutated ubiquitin leading to the expression of ubiquitin-DNA fusion proteins that were efficiently degraded in the proteasome. T cells recognized the TH self-antigen epitopes indicating that the self-tolerance against TH can be overcome with this approach [143]. 

In subsequent studies the same mTH-based minigenes, novel epitopes, and xenogeneic TH DNA vaccination were effective in therapeutic settings to suppress established neuroblastoma metastases. Modifications of mTH had additional positive effects. The mutated ubiquitin of the used plasmid was crucial for the strong antitumoral effect leading to a CD8^+^ T cell immune response. Primary tumors were infiltrated by CD8^+^ T cells, and TH-expressing cells were specifically lysed *in vitro*. Depletion of CD8^+^ T cells completely abrogated the anti-NB immune response induced by the hTH vaccine. Rechallenge of surviving mice resulted in reduced primary tumor growth, indicating the induction of a memory immune response. An important observation was that immunization with the self-antigen TH did not lead to autoimmunity [18, 144, 145]. 

In a different study using the same mouse model, novel natural MHC class I ligands from neuroblastoma were characterised and used in a DNA minigene approach. Immunization of mice induced protective immunity and thus underlines the assumption that disruption of self-tolerance against neuroblastoma-associated epitopes is important for an effective neuroblastoma immunotherapy [146]. 

The inhibitor of apoptosis protein (IAP) survivin is highly expressed in neuroblastoma and is associated with a poor prognosis. Therefore, survivin was chosen as a target for NB DNA vaccination. A survivin DNA minigene efficiently inhibited the growth of primary tumor and metastases in the NXS2 tumor model. The used DNA minigene was as effective as a survivin full-length cDNA vaccine showing the power of DNA minigene vaccination. Immunization with survivin minigene was associated with an increased presence of CD8^+^ T cells in the primary tumor and production of the proinflammatory cytokines INF-*γ* and TNF-*α* by systemic CD8^+^ T cells. Depletion of CD8^+^ but not CD4^+^ cells led to a complete abrogation of the tumor immunity. Therapeutic vaccination with the minigene was able to eradicate neuroblastoma in more than half of the mice, and surviving mice were protected from primary tumor growth after rechallenge with tumor cells [147].

In summary, these preclinical studies suggest that DNA vaccination using *S. thyphimurium *as a delivery system may provide an important strategy to develop an active immunotherapy strategy for this challenging disease.

## 4. Prostate Cancer

Prostate cancer is the most common cancer and the fourth leading cause of cancer-related death in men in the developed countries worldwide [1, 88, 148]. Standard prostate cancer therapy in early diagnosed patients involves prostatectomy, cryotherapy, radiotherapy, and antiandrogen therapy. These treatments are effective but bear the risk of severe side effects like incontinence and impotence [149–151]. Therefore, there is an urgent need for novel approaches to treat this disease.

CD4^+^ and CD8^+^ T cells are detectable in prostate glands of men with prostate cancer supporting the assumption that prostate cancer might be a good candidate for immunotherapy. Prostate cancer cells are usually growing rather slow, permitting enough time to use vaccination as an approach to overcome immunosuppressive factors [43]. Currently, there are several clinical trials using immunotherapy against prostate cancer targeting prostate cancer-associated antigens including Prostate-Specific Antigen (PSA) [152], Six-Transmembrane Epithelial Antigen of the Prostate (STEAP) [153], Prostate Stem Cell Antigen (PSCA) [154], Prostate-Specific Membrane Antigen (PSMA) [54, 155], and Prostatic Acid Phosphatase (PAP) [156]. 

PSMA was one of the first prostate cancer-associated antigens used for DNA vaccination. Plasmid DNA and adenoviral vectors encoding for PSMA were used to immunize patients with prostate cancer in a phase I/II trial. Costimulation of plasmid DNA with the molecule CD86 led to delayed-type hypersensibility to PSMA in half of the patients, but additional boosting with the adenovirus was necessary to induce immunity in all of them. However, the success of this study is difficult to interpret due to the heterogeneity of the patients, and the concomitant hormone therapy many of the patients received albeit local disease, distant metastases, and PSA levels changed positively [39]. In another study, patients were vaccinated against PSMA with plasmid DNA and adenovirus as well leading to the detection of specific anti-PSMA antibodies in the sera of the patients [157]. A recent clinical phase I/II demonstrated the power of electroporation in induction of a humoral immune response against prostate cancer. PSMA-specific DNA vaccines were delivered by intramuscular injection or in combination with an additional delivery by electroporation (EP). The boosting by EP significantly enhanced (24.5-fold increase) the immune response during an 18-month followup period [54].

In summary, the efforts made in clinical trials using DNA vaccination against prostate cancer are promising, but the response rates have to be improved. Therefore, the evaluation and characterization of DNA vaccination strategies in preclinical models is an important venue. The following paragraphs summarize some of the research in this respect.

One approach is the use of xenogeneic vaccination strategies. The effectiveness of mouse PSMA and human PSMA (hPSMA) DNA vaccines were tested in an animal model indicating that only xenogeneic hPSMA was able to induce both antibody as well as T cell responses against the murine self-antigen. The antibodies induced were able to recognize the human and the murine PSMA, and it was concluded that xenogeneic DNA is a requirement to overcome the immunologic tolerance against the poorly immunogenic PSMA in contrast to other studies [158]. These results were improved in a setting with immunization of mice using xenogeneic hPSMA DNA followed by boosting with hPSMA protein [17]. 

Further improvements can be achieved by modifications of DNA vaccines leading to expression and proteasomal degradation of hPSMA in combination with protein boosting. This resulted in antibody formation of the cytotoxic Th 1 isotypes, and the best protection against tumor challenge was observed after immunization with the xenogeneic hPSMA construct following boosting with the syngeneic construct [159]. 

The use of cytokines in order to amplify suboptimal immune responses following Prostate-Specific Antigen (PSA) DNA vaccination is another strategy to increase DNA vaccine efficacy. A DNA vaccine expressing PSA induced PSA-specific CTLs when coinjected with the costimulatory cytokines IL-2 and GM-CSF and protected the majority of immunized mice against a lethal tumor challenge [160]. 

Also, the delivery system is an important factor to induce effective immunity against prostate cancer. Intradermal immunization of mice with PSA induced strong humoral and cellular immune responses of the Th 1 isotype indicated by strong expression of INF-*γ* and IL-2 and protected mice from challenge with PSA-expressing tumor cells [161]. Intramuscular electroporation with human PSA (hPSA) DNA significantly reduced tumor growth and increased the survival of mice after a lethal challenge with hPSA-expressing TRAMP-C1 cells, a cell line developed from a prostate tumor of a TRAMP (transgenic adenocarcinoma mouse prostate) mouse. Production of hPSA-specific antibodies and expression of IFN-*γ* was observed in the immunized animals [51]. Multiple CTL and T-helper cell epitopes of hPSMA, mPAP, and hPSA were combined to generate a DNA vaccine that should have a stronger effect against prostate tumor cells than single antigen vaccines. The vaccine design was chosen to overcome the problem that tumor cells often lose antigenic epitopes and escape immunologic detection. Vaccination of mice by gene gun induced a strong immune response against applied tumor cells and increased the survival time significantly [162]. A systematic comparison with other delivery systems including life-attenuated bacteria was not reported so far.

Other antigens investigated in preclinical models are the Prostatic Acid Phosphatase (PAP) and Prostate Stem Cell Antigen (PSCA). PAP is effective in inducing proliferating PAP-specific CD4^+^ and CD8^+^ T cells of the Th 1 isotype and expression of IFN-*γ* in rats immunized with a human PAP DNA vaccine [163, 164]. Application of a PAP DNA vaccine to prostate cancer patients induced a PAP-specific T cell response and showed no side effects making the use of PAP in clinical stage II trials likely [165]. Vaccination of mice with plasmid encoding for human PSCA induced strong PSCA-specific CD8^+^ T cell responses and inhibited the growth of PSCA-positive tumors [166]. There are no comparative studies allowing a decision about which antigen might be the best choice.

The models used to study DNA vaccination in prostate cancer are usually based on the TRAMP model. In TRAMP mice, the Simian virus 40 (SV40) tumor T-antigens are prostate-specifically expressed driving prostate neoplasia, which results in poorly differentiated adenocarcinomas of the prostate and metastasis in lung and pelvic lymph nodes resembling the human disease [167, 168]. TRAMP mice were strongly protected against tumor development when vaccinated with PSCA-based DNA, an effect supposedly mediated by CD8^+^ T cells and expression of the cytokines INF-*γ*, TNF-*α*, IL-2, IL-4, and IL-15 within prostate tumors. Importantly long-term protection was not accompanied by an induction of autoimmunity [169]. From this mouse model, TRAMP-C1 tumor cells were isolated which grow in syngeneic C57BL/6 mice. In this model, vaccination with PSCA DNA by intramuscular electroporation induced an effective antitumor response, and the mice were either cured or showed a significant increase in survival, which was mediated by an immunity of the Th 1 type [170]. 

An interesting discovery was made when two therapeutic vaccination studies with the antigens PSCA and STEAP in the TRAMP mouse model were compared. DNA vaccination at an early stage of disease resulted in an improved protection against tumor development and an increased survival time when compared to vaccination after the development of invasive carcinoma. Regulatory  T cells as well as the expression of immunosuppressive factors like TGF-*β* and indoleamine-2,3-dioxygenase were detected in more advanced prostate cancer making the use of DNA vaccination at earlier stages of disease more promising [171].

In summary, DNA vaccination against prostate cancer has demonstrated effectiveness in preclinical models, and promising immune responses were observed in early clinical trials. Given the amount of preclinical information on selection and design of suitable and effective antigens, a system biology approach may provide an important venue to translate available information into an effective immunotherapy.

## 5. Summary

DNA vaccination is a young field in immunotherapy of cancer and has certainly not yet lived up to its expectations. However, considering the fact that the development of antibodies into effective cancer therapeutics followed a timeline of over a century, DNA vaccination may be considered to be on a fast track development. Preclinical data are very promising and significant immune responses can be demonstrated in several clinical trials especially in the field of melanoma DNA vaccination. The high versatility, the ease of production, and the stability of DNA vaccines may provide important characteristics to further develop this approach into effective cancer therapies of the 21st century.
